# The characteristics and risk factors for common psychiatric disorders in patients with cancer

**DOI:** 10.1192/j.eurpsy.2023.2006

**Published:** 2023-07-19

**Authors:** T. Jupe, K. Provi, I. Giannopoulos, B. Zenelaj, E. Myslimi

**Affiliations:** 1 Psychiatric Hospital of Attica, Athens, Greece; 2 National Center for Children Treatment and Rehabilitation; 3 Freelancer psychiatrist, Tirane, Albania

## Abstract

**Introduction:**

The incidence of psychological disorders in patients with cancer is very high (30–60%), with approximately 29–43% fulfilling the diagnostic criteria for psychiatric disorders. The most commonly encountered mental problems encompass depressive symptoms associated with mixed anxiety and adjustment disorder or depressive mood or major depression.

**Objectives:**

The aim of this research is to highlight the characteristics of psychiatric manifestations in patients with cancer and to analyse the risk factors that influence the occurrence of these psychiatric manifestations.

**Methods:**

Α bibliographical review was performed using the PubMED platform. All relevant articles were found using the keywords: cancer, psychiatric manifestations, risk factors.

**Results:**

Sleep problems, irritability, tendency to cry easily, sadness, and pain were among the leading symptoms at baseline. Women reported sleep problems, tendency to cry easily, irritability, pre-occupation with the illness, and sadness as the first five most frequent issues, and men reported sleep problems, irritability, pain (usually incompatible with their medical conditions), sadness, and tendency to cry easily as the most frequent problems.

**Conclusions:**

Significant risk factors that increased the mood disorders were recurrence, presence of secondary cancer, other chronic medical illnesses, history of psychiatric disorder, low income level, poor social support, and being single or divorced.

**Disclosure of Interest:**

None Declared

